# Aptamer-Aptamer Chimera for Targeted Delivery and ATP-Responsive Release of Doxorubicin into Cancer Cells

**DOI:** 10.3390/ijms222312940

**Published:** 2021-11-30

**Authors:** Ezaldeen Esawi, Walhan Alshaer, Ismail Sami Mahmoud, Dana A. Alqudah, Bilal Azab, Abdalla Awidi

**Affiliations:** 1 Faculty of Medicine, The University of Jordan, Amman 11942, Jordan; ezaldeenesawi@gmail.com (E.E.); azab.belalm@gmail.com (B.A.); 2 Cell Therapy Centre, The University of Jordan, Amman 11942, Jordan; pharmd.dana.alqudah@gmail.com; 3 Department of Medical Laboratory Sciences, Faculty of Applied Medical Sciences, The Hashemite University, Zarqa 13133, Jordan; ismails@hu.edu.jo; 4 Department of Hematology and Oncology, Jordan University Hospital, The University of Jordan, Amman 11942, Jordan

**Keywords:** aptamers, drug delivery, chimera, targeted delivery, nucleolin, stimuli-responsive release, doxorubicin

## Abstract

Aptamers offer a great opportunity to develop innovative drug delivery systems that can deliver cargos specifically into targeted cells. In this study, a chimera consisting of two aptamers was developed to deliver doxorubicin into cancer cells and release the drug in cytoplasm in response to adenosine-5′-triphosphate (ATP) binding. The chimera was composed of the AS1411 anti-nucleolin aptamer for cancer cell targeting and the ATP aptamer for loading and triggering the release of doxorubicin in cells. The chimera was first produced by hybridizing the ATP aptamer with its complementary DNA sequence, which is linked with the AS1411 aptamer via a poly-thymine linker. Doxorubicin was then loaded inside the hybridized DNA region of the chimera. Our results show that the AS1411–ATP aptamer chimera was able to release loaded doxorubicin in cells in response to ATP. In addition, selective uptake of the chimera into cancer cells was demonstrated using flow cytometry. Furthermore, confocal laser scanning microscopy showed the successful delivery of the doxorubicin loaded in chimeras to the nuclei of targeted cells. Moreover, the doxorubicin-loaded chimeras effectively inhibited the growth of cancer cell lines and reduced the cytotoxic effect on the normal cells. Overall, the results of this study show that the AS1411–ATP aptamer chimera could be used as an innovative approach for the selective delivery of doxorubicin to cancer cells, which may improve the therapeutic potency and decrease the off-target cytotoxicity of doxorubicin.

## 1. Introduction

The coining of the “magic bullet” concept inspired generations of researchers to develop treatments with reduced toxicity and enhanced therapeutic efficacy. Based on this concept, targeted therapies and targeted drug delivery systems have been developed for the selective targeting of diseased tissues such as tumors [1,2,3]. Over the last few decades, targeted delivery systems have offered a new arsenal for more efficient and safer cancer treatments, due to reduced toxicity and improved bioavailability [4]. Various biological ligands have been used for active targeting therapy, such as antibodies, peptides, and aptamers [5,6].

Aptamers are short single-stranded DNA or RNA screened via Systematic Evolution of Ligands by EXponential enrichment (SELEX) [3]. Their unique tertiary structure enables them to specifically bind to a large variety of target molecules. Therefore, they have been widely used as ligands for active targeting therapy [7]. Aptamers are characterized by their low immunogenicity, structural stability, and flexibility, in addition to their high affinity and ease of synthesis [8]. Using an aptamer as a targeted delivery ligand, which can selectively and efficiently deliver therapeutics to target cells, is expected to significantly reduce systemic toxicity and/or improve the therapeutic effectiveness of drugs [3]. Many aptamers, such as the AS1411 aptamer, have been used as targeting ligands [9]. The AS1411 DNA aptamer specifically recognizes nucleolin receptors, a protein which is overexpressed in breast cancer and glioblastoma [10,11], which has been used as a targeting moiety in many drug delivery systems, such as liposomes and gold nanoparticles [12,13]. The chemistry of aptamers makes it possible to easily generate multivalent structures from aptamers to achieve various goals in diagnosis and therapy [14]. Liu et al. [15] provide a recent example. The authors designed a bispecific RNA–aptamer chimera able to target two tumor markers, CD44 and epithelial cell adhesion molecules (EpCAM), combined through a double-stranded RNA adaptor. This chimera resulted in an increase in circulatory half-life, thus a more efficient inhibition of cell growth. Another innovative example highlighting the great potential of aptamer chimeras was reported by Shigdar et al. [16]. They addressed the delivery of an EpCAM aptamer to the brain by employing a second aptamer that targets the transferrin receptor, which allows receptor-mediated transcytosis of the ligand through the blood–brain barrier.

Currently, there are tremendous efforts being directed at exploring active targeting and stimuli-responsive drug delivery systems that stimulate drug release “on demand” in a spatiotemporally controlled fashion to further enhance their biological specificity and therapeutic efficacy [17].

**Figure 1:**
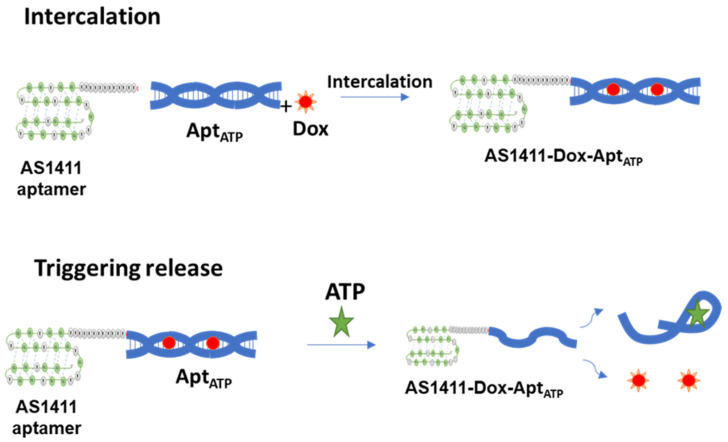
Design of the generated aptamer–aptamer chimera.

In this study, we generated an aptamer–aptamer chimera that comprises an AS1411 anti-nucleolin aptamer for targeted delivery and an ATP aptamer loaded with doxorubicin for stimuli-triggering release (Figure 1). The targeting and responsive releases of the doxorubicin-loaded chimera were evaluated in cancer and normal cells in vitro. The described approach extends the potential utility of aptamer-based drug delivery systems by allowing the creation of novel combinatorial strategies with increased selectivity and therapeutic efficacy.

## 2. Results and Discussions

### 2.1. The Successful Generation of AS1411–ATPapt Chimera Loaded with Doxorubicin

**Figure 2:**
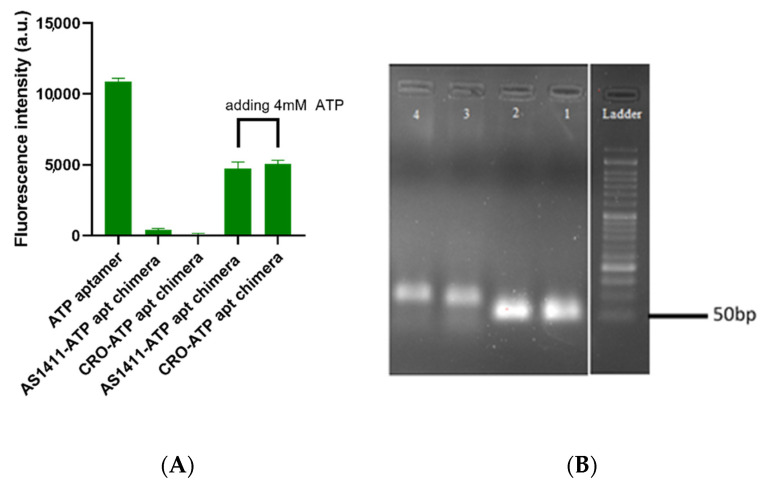
Detection of annealing of ATP aptamer with its complementary DNA, and the ATP-triggering dissociation of ATP aptamer from its complementary DNA strand. (**A**) Quenching-based analysis was used for annealing detection, and ATP triggering dissociates the ATP aptamer from its complementary DNA strand. Error bars indicate SD. (*n* = 3). (**B**) Gel electrophoresis assay confirms that ATP aptamer hybridized with its complementary DNA. Lane 1: Unannealed DNA strand contains AS1411 aptamer, lane 2: Unannealed DNA strand contains CRO, lane 3: formed AS1411–ATP aptamer chimera, lane 4: formed CRO–ATP aptamer chimera.

We successfully constructed the chimera (AS1411–ATPapt) that contains a loading domain for doxorubicin by annealing the ATP DNA aptamer and its complementary DNA strand, which has been linked to the AS1411 aptamer, for specific targeting of the chimera. After this, the control chimera CRO–ATPapt was generated, where the AS1411 aptamer was replaced with a control sequence for non-specific targeting. Figure 2A presents the annealing step between the ATP DNA aptamer and its complementary DNA sequence, which was confirmed by fluorescence quenching. This was detected between the Cy5 dye conjugated to ATP aptamer and the Iowa black RQ quencher (common quencher for Cy5) conjugated to the complementary DNA sequence. In fact, it was clearly observed that most of the Cy5 fluorescence was quenched upon the formation of the chimera. In addition, Figure 2B demonstrates the formation of annealed chimeras based on the size of the bands on the agarose gel, using gel electrophoresis. Following this, to evaluate the chimera disassembly in response to (adenosine triphosphate) ATP, the chimeras were incubated with 4 mM ATP molecules for 15 min in vitro, and fluorescence quenching and recovery of measurements were used for investigation. The chimeras showed an excellent response to ATP molecules, as inferred by the recovery of the fluorescence intensity of Cy5 in the presence of ATP, which was about 45% (Figure 2A). We suppose that a conformational change in ATP aptamer occurred upon binding with the ATP. This conformational change caused the recovery of the Cy5 fluorescent that was connected with the ATP aptamer.

**Figure 3:**
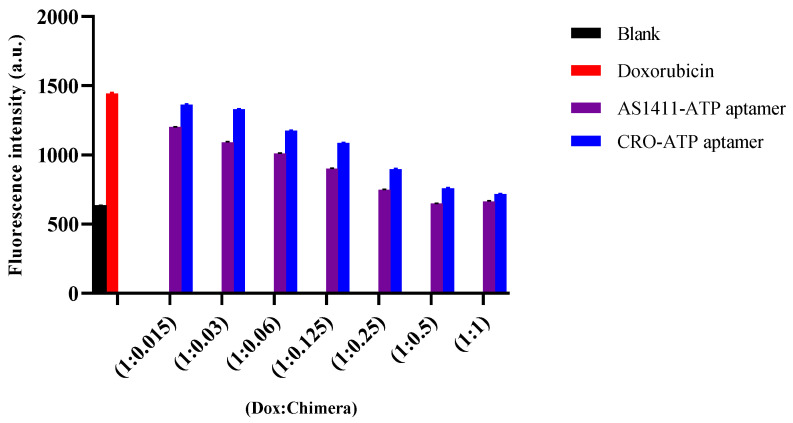
Fluorescence intensity of doxorubicin with increasing molar ratio of each chimera. Doxorubicin loading efficiency was measured by monitoring the fluorescence intensity of doxorubicin when incubated with a different molar ratio of each chimera. A fixed concentration of doxorubicin (2 μM), dissolved in nuclease-free water, was incubated with a different molar ratio of the chimeras and then incubated for 15 min. The fluorescence intensity of doxorubicin was measured using Glomax microplate reader. Blank is the working buffer. Error bars indicate SD (*n* = 3).

In a similar way, doxorubicin loading efficiency was measured by monitoring the fluorescence intensity of doxorubicin when incubated with a different molar ratio of each chimera. The fluorescence intensity of doxorubicin decreased sequentially, due to intercalation into the chimera. The maximum quenching of doxorubicin fluorescence in both AS1411–ATPapt and CRO–ATPapt (~100% and 90%, respectively) was achieved at a molar ratio of 1:0.5 (doxorubicin: chimera), as shown in Figure 3.

### 2.2. ATP-Responsiveness for Doxorubicin Release from the Generated Chimeras

**Figure 4:**
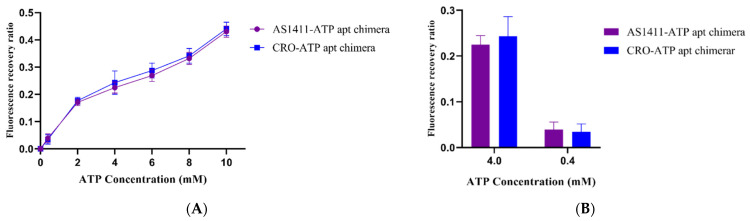

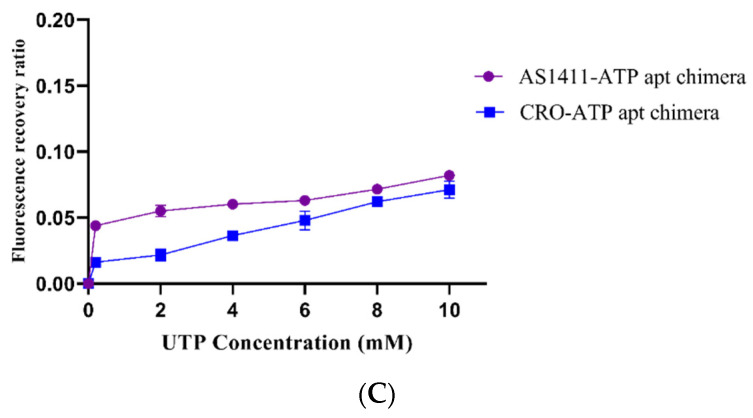
ATP-triggered doxorubicin release from the aptamer–aptamer chimera. (**A**) Fluorescence recovery ratios of aptamer–aptamer Dox loaded in the presence of different concentrations of ATP. Error bars indicate SD (*n* = 3). (**B**) Fluorescence recovery ratios of doxorubicin-loaded chimeras in the presence of 4 mM ATP and 0.4 ATP. Error bars indicate SD (*n* = 3). (**C**) Fluorescence recovery ratios of doxorubicin-loaded chimeras in the presence of different concentrations of UTP. Error bars indicate SD (*n* = 3).

The doxorubicin-loaded chimeras were incubated with different concentrations of ATP or UTP (uridine triphosphate), and then the doxorubicin fluorescence intensity was monitored. The chimeras exhibited a selectivity in binding between ATP and UTP for releasing the loaded doxorubicin. Dissociation of the ATP aptamer from the chimera after adding ATP led to the release of loaded doxorubicin (Figure 4). The amount of doxorubicin released was estimated by calculating the fluorescence recovery ratio, as shown in Figure 4A. The fluorescence recovery ratio of doxorubicin from the chimeras was tested against different concentrations of ATP. Indeed, in the presence of 4 mM concentration ATP, which is equal to the intracellular concentration of ATP, the fluorescence recovery ratio was about four-fold greater than that of 0.4 mM concentration ATP, which resembles the extracellular ATP concentration (Figure 4B). Interestingly, insignificant changes in the fluorescent recovery ratio of doxorubicin were observed after incubation of the chimeras with UTP (Figure 4C), indicating the selective response of the chimera to ATP molecules.

### 2.3. The Stability of Doxorubicin-Loaded AS1411–ATPapt Chimera in Serum

**Figure 5:**
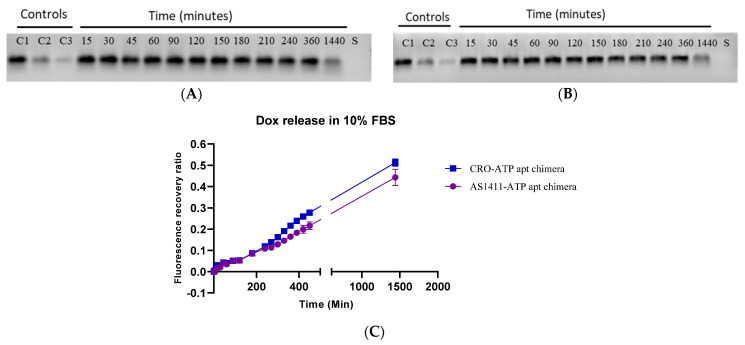
Serum stability assay of the generated chimeras. (**A**) Serum stability of AS1411–ATP chimera on gel electrophoresis. (**B**) Serum stability of CRO–ATP chimera on gel electrophoresis. The bands of chimera are shown in black in 2% agarose gel. The numbers at the top indicate the time of incubation with serum in minutes. C1 = 1 µg of chimera, C2 = 0.5 µg and C3 = 0.25 µg used as control, S = serum blank. (**C**) Fluorescence recovery ratio of doxorubicin from both chimeras after incubation with 10% FBS.

The stability of doxorubicin-loaded AS1411–ATPapt chimera was tested in 10% fetal bovine serum (FBS), which contains several nucleases [18]. The chimera was incubated with the FBS for different periods of time, then an agarose gel electrophoresis assay was used to visualize the chimera bands. Strikingly, the chimeras were stable for several hours (Figure 5A,B). Moreover, the intercalation of doxorubicin inside the chimera was stable, as proved by the low fluorescence recovery ratio of doxorubicin after incubation in 10% FBS for several hours (Figure 5C).

### 2.4. Cytotoxicity of AS1411–ATPapt Chimera in Cancer Cells

**Figure 6:**
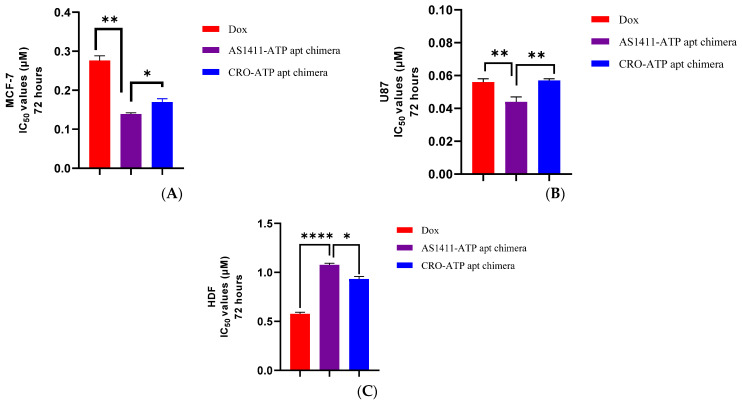
IC_50_ values after 72 h of incubation of the chimeras with cell lines. (**A**) MCF-7 cell line. (**B**) U87 cell line. (**C**) HDF cell line. Error bars indicate SD (*n* = 3). * *p* < 0.05, ** *p* < 0.01, **** *p* < 0.0001 (one-way ANOVA).

Viability assays were conducted in vitro to investigate the efficiency of the AS1411–ATPapt chimera against MCF-7, U87, and HDF cell lines. Cell lines were treated with increased concentrations of doxorubicin-loaded chimeras for 72 h. Cells treated with free doxorubicin and CRO–ATPapt were also included in the assays for comparison. The IC_50_ for each treatment was calculated, as shown in Figure 6. In U87 and MCF-7 cell lines, the doxorubicin-loaded AS1411–ATPapt chimera showed a statistically significant lower IC_50_ value compared with the free doxorubicin and the CRO–ATPapt chimera (Figure 6A,B), indicating the higher cytotoxic activity of AS1411–ATPapt in cancer cells. Prominently, both AS1411–ATPapt and CRO–ATPapt chimeras showed less toxic effects in the HDF cell line compared with free doxorubicin (Figure 6C).

**Figure 7:**
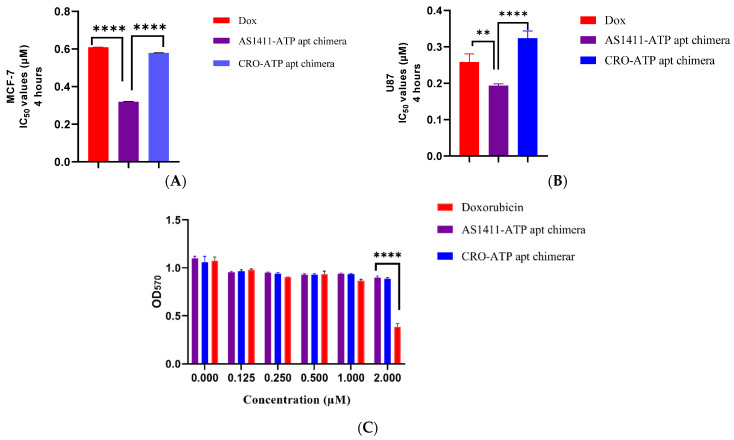
IC_50_ values after 4 h of incubation of the chimeras with cell lines. (**A**) MCF-7 cell line. (**B**) U87 cell line. (**C**) HDF cell line. Error bars indicate SD (*n* = 3). ** *p* < 0.01, **** *p* < 0.0001 (one-way ANOVA).

To further examine the active targeting of the chimeras and the effect on cytotoxic activity, the cells were treated with doxorubicin, AS1411–ATPapt, and CRO–ATPapt for only 4 h and then replaced with a fresh culture medium. The viability assay was conducted after 72 h, and the results show selective uptake of the AS1411–ATPapt chimera targeted via nucleolin aptamer in both MCF-7 and U87 cells (Figure 7). The IC_50_ results of the AS1411–ATPapt chimera are ~2-fold lower than CRO–ATPapt chimera and free doxorubicin in MCF-7 cells (Figure 7A) and about 1.6-, 1.3-fold lower than the CRO–ATPapt chimera and free doxorubicin in U87 cells, respectively (Figure 7B). These differences in cytotoxicity might be explained by the cellular uptake mechanism of each treatment. As for the AS1411–ATPapt chimera uptake, the presence of the AS1411 aptamer would mediate the targeting of the nucleolin receptors on cancer cells and subsequent cellular uptake of the chimera through macropinocytosis [12]. In fact, the cellular uptake of nucleolin aptamer through macropinocytosis has been reported previously, where Reyes et al. showed that nucleolin aptamers could hyper-stimulate macropinocytosis in cells [19], which may explain the increase in the uptake of the AS1411–ATPapt chimera and induction of cell death compared with the CRO–ATPapt chimera and free doxorubicin [19,20]. Notably, that chimeras showed very low toxicity on the HDF cell line compared to free doxorubicin (Figure 7C). Overall, these results show that the AS1411–ATPapt chimera loaded with doxorubicin has high anti-cancer activity but low toxicity on normal cells compared to free doxorubicin.

### 2.5. The AS1411–ATPapt Chimera Is Efficiently Taken up by Cells

**Figure 8:**
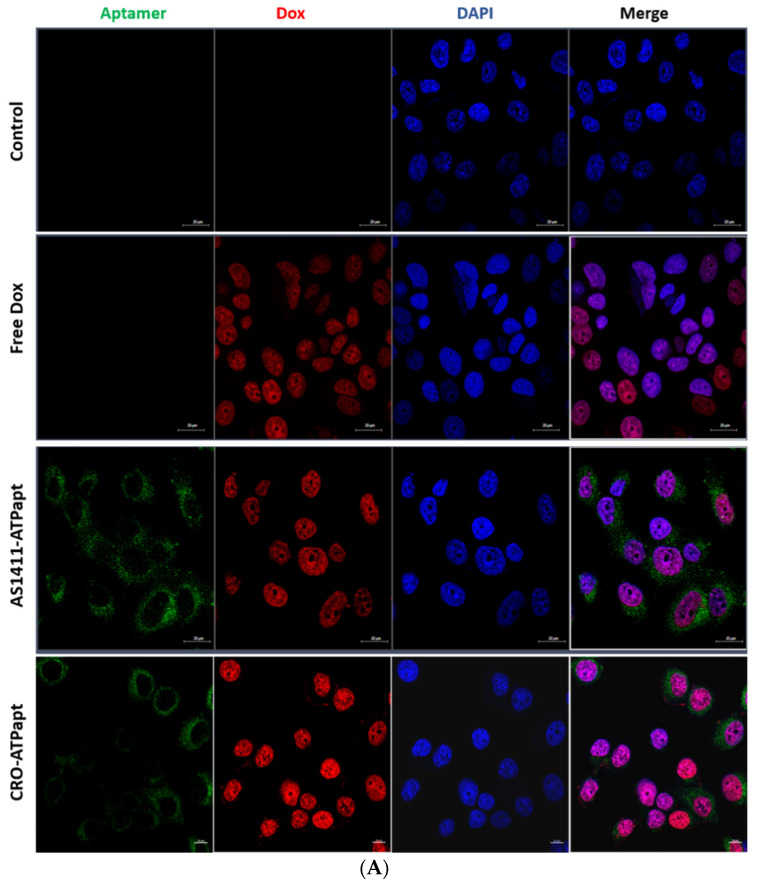

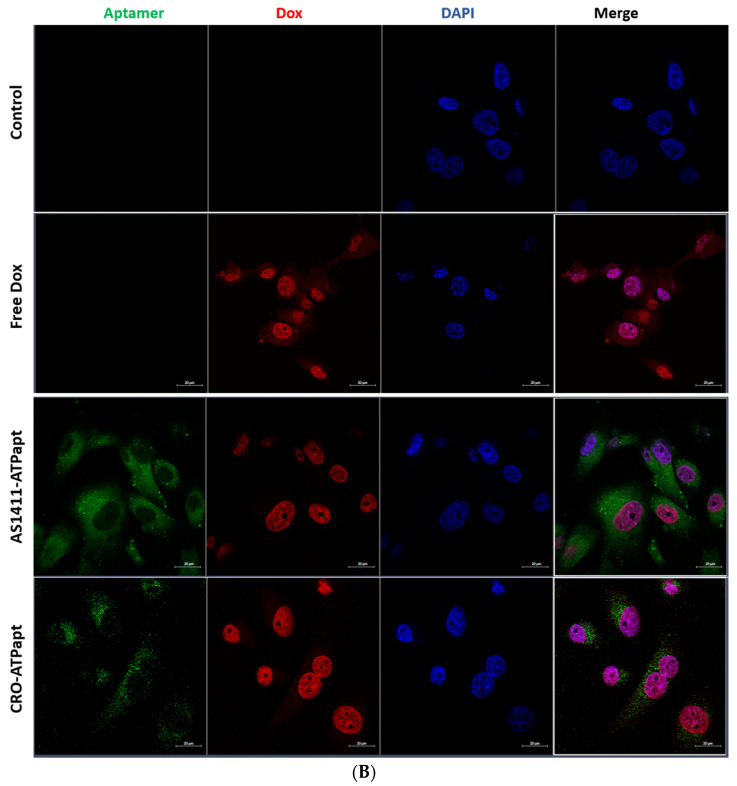
Cellular uptake of chimeras demonstrated by confocal microscopy. The cancer cells (**A**) MCF-7 and (**B**) U87 were incubated with a culture medium containing free doxorubicin or doxorubicin loaded into fluorophore-labeled chimeras. For negative control, cells were incubated with complete culture medium without treatment. After incubation at 37 °C for 3 h, the cells were fixed in 4% formaldehyde and then analyzed by confocal laser microscope (Dox showed red fluorescence, Cy5-labeled ATP aptamer showed green fluorescence, and the nuclei stained with DAPI showed blue fluorescence).

The cellular uptake of doxorubicin-loaded chimeras was defined using confocal laser scanning microscopy. The confocal microscopy analysis based on the Cy5 fluorescence signal demonstrates that both the AS1411–ATPapt and CRO–ATPapt chimeras were taken up by cells (Figure 8). The uptake of AS1411–ATPapt was very prominent in in both U87 and MCF-7 cancer cell lines, whereas only little of the CRO–ATPapt chimeras was observed in the cancer cell lines (Figure 8). Notably, a punctate endosomal-like distribution of the Cy5–aptamer fluorescence signal in the cells was observed, which probably indicates a cytoplasmic localization of the chimera (Figure 8). It is worth mentioning that the entry of the aptamers into cells can be mediated by either clathrin-dependent or calthrin-independent pathways, depending on their targets [21], and so the internalization of the AS1411–ATPapt and CRO–ATPapt chimeras could be through different pathways, which may explain the difference in uptake efficiency and cytotoxicity between the two chimeras. On the other side, the doxorubicin released from both AS1411–ATPapt and CRO–ATPapt chimeras was mostly localized in the nucleus of the cancer cell lines (Figure 8), which indicates the ability of the generated chimeras to deliver the doxorubicin to its correct subcellular location.

**Figure 9:**
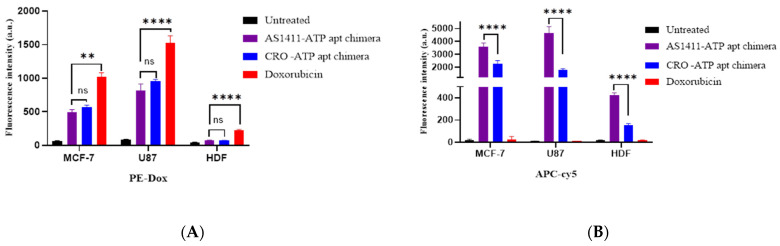
FACS analysis of cellular uptake of the generated chimeras in MCF-7, U87, and HDF after incubation with free doxorubicin and doxorubicin loaded in chimeras. (**A**) Fluorescence intensity of doxorubicin. (**B**) Fluorescence intensity of Cy5. Error bars indicate SD (*n* = 3). ** *p* < 0.01, **** *p* < 0.0001 (one-way ANOVA).

Moreover, quantitative cellular uptake was investigated by flow cytometry based on the recovery of Cy5 fluorescence and fluorescence intensity of doxorubicin upon chimeras internalized into the cells and dismantled. Flow cytometry performed on MCF-7, U87, and HDF cell lines showed successful uptake and disassembly of the chimeras. Figure 9 demonstrates the fluorescence intensity of doxorubicin and Cy5 in cells after uptake of the chimeras measured by flow cytometry. Interestingly, the AS1411–ATPapt chimera showed a higher uptake in MCF-7 and U87 cells as compared with the CRO–ATPapt chimera, indicated by the higher fluorescence intensity of Cy5 in cells (Figure 9A). In addition, the AS1411–ATPapt chimera had a selective uptake into cancer cells presented by the significantly higher uptake in cancer cell lines compared to HDF cell line (Figure 9A). On the other hand, there was no significant statistical difference in the fluorescence intensity of the doxorubicin released from the AS1411–ATPapt and CRO–ATPapt chimeras (Figure 9B), even though the uptake of the AS1411–ATPapt chimera was higher in the cells. Theoretically, the amount of doxorubicin released in cells should be proportional to the amount of chimera uptake; however, we did not observe any significant difference in doxorubicin uptake between the AS1411–ATPapt and CRO–ATPapt chimeras, which could be attributed to the fluorescence characteristics of doxorubicin. Indeed, it was demonstrated that the fluorescence lifetime of doxorubicin in the nucleus decreases rapidly during the first 2 h following drug administration [22]. Thus, monitoring the dynamics of doxorubicin fluorescence lifetimes using other techniques such as fluorescence lifetime imaging microscopy (FLIM) [22] may provide more valuable information about the quantity of doxorubicin localized in the cells, particularly during the earliest phases of doxorubicin administration.

## 3. Materials and Methods

### 3.1. Chemicals, Reagents, and Cell Lines

All nucleic acid sequences, including the fluorophore-labeled sequences, were purchased from Integrated DNA Technologies (San Diego, CA, USA) (sequences are provided in Table 1). Doxorubicin HCl was purchased from Sigma-Aldrich (Burlington, MA, USA). Adenosine triphosphate (ATP) and uridine triphosphate (UTP) from Santa Cruz Biotechnology (Dallas, TX, USA). Ethidium bromide from Bio Basic (Markham, ON, Canada). RPMI 1640 Medium, penicillin, streptomycin, L-Glutamine, and trypsin–EDTA from EuroClone, (Milan, Italy). DAPI from Thermofisher (Waltham, MA, USA). Dulbecco’s Modified Eagle Medium from Gibco (Waltham, MA, USA). Fetal bovine serum from Capricorn Scientific (Ebsdorfergrund, Germany). 3-(4,5-Dimethyl-2-thiazolyl)-2,5-diphenyltetrazolium bromide (MTT) salts from Bioworld (Visalia, MO, USA). Agarose gel from Conda Lab (Madrid, Spain). MCF-7, U87, and HDF cell lines were obtained from ATCC (Manassas, VA, USA).

### 3.2. Design and Formation of Aptamer–Aptamer Chimeras

Two chimeras were generated. The first chimera was called AS1411–ATPapt, which consisted of the anti-nucleolin AS1411 aptamer (5′-GGTGGTGGTGGTTGTGGTGGTGGTGG) linked via poly-thymine to the complementary DNA strand that annealed specifically to the ATP DNA aptamer. The second chimera, CRO–ATPapt, which was composed of the control, the cytosine-rich sequence (5′-CCTCCTCCTCCTTCTCCTCCTCCTCC) linked via poly-thymine to the complementary DNA strand that annealed specifically to the ATP DNA aptamer. For imaging experiments, we used the following fluorophore-labeled sequences: Iowa Black RQ-labeled complementary DNA strand and the ATP DNA aptamer labeled by Cy5 fluorophore (Table 1). The Iowa Black RQ was used as a quencher to block the emission of Cy5 fluorophore when the two are close together. Chimeras were prepared by mixing the ATP aptamer and its complementary DNA strand at a molar ratio of 1:1 in a working buffer (5 mM HEPES buffer containing 10 mM MgCl2 and 137 mM NaCl), then incubating at 90 °C for 2 min, followed by 1 min at 70 °C, 1 min at 65 °C, 1 min at 60 °C, and 10 min at 4 °C. To assess the formation of the chimeras, 1 µg from the annealed sequences and 0.5 µg from the non-annealed sequences were diluted with 10 μL of nuclease-free water, then mixed with DNA loading dye, and then loaded into a 2% agarose gel containing 1 μg/mL ethidium bromide. The gel was run at 70 V for 35 min. The chimera’s band was visualized using Bio-Rad’s ChemiDoc MP imaging system and Image Lab software.

### 3.3. Evaluate ATP Responsiveness of the Aptamer–Aptamer Chimeras

To evaluate the response of the generated chimeras to the ATP molecule and subsequent disconnection of the ATP aptamer, the fluorophore-labeled chimeras were incubated with 4 mM of ATP molecules in 75 µL of working buffer at 37 °C for 25 min, covered in foil, then the fluorescence intensity of Cy5 fluorophore was measured using a Glomax microplate reader (Promega, Madison, WI, USA). The fluorescence intensity of Cy5 was expected to increase as the ATP aptamer separated from its complementary sequence containing the quencher Iowa Black RQ.

### 3.4. Doxorubicin Loading into the Aptamer–Aptamer Chimeras

A fixed concentration of doxorubicin (2 μM), dissolved in nuclease-free water was incubated with a different molar ratio of the chimeras (0, 0.01, 0.025, 0.05, 0.1, 0.25, 0.5, and 1 equivalent) in the working buffer and then incubated for 15 min covered in foil. The fluorescence intensity of doxorubicin was measured using a Glomax microplate reader (Promega, Madison, WI, USA), with an excitation peak measured at 490 nm and an emission peak at 510–570 nm. The fluorescence intensity of doxorubicin was expected to decrease as more doxorubicin intercalated into DNA.

### 3.5. Triggering the Release of Doxorubicin from the Aptamer–Aptamer Chimeras

To investigate the ability of doxorubicin-loaded chimeras to release doxorubicin specifically in response to ATP, each chimera loaded with doxorubicin (at a molar ratio of 1:2, respectively) was incubated with different concentrations of ATP and UTP (10, 8, 6, 4, 2, 0.4, and 0 mM) in the working buffer at 37 °C for 25 min, covered in foil. After that, the fluorescence recovery of doxorubicin was measured using a Glomax microplate reader (Promega, Madison, WI, USA), an excitation peak at 490 nm and an emission peak at 510–570 nm were measured. The following equation was used to calculate the fluorescence recovery of doxorubicin:

Fluorescence Recovery Ratio = ((F_NTP_ − F) ÷ (F_0_ − F))

where F_NTP_ and F are the fluorescence intensities of doxorubicin in chimeras in the presence and absence of ATP, respectively, and F_0_ is the fluorescence intensity of free doxorubicin.

### 3.6. In Vitro Serum Stability Assay of Chimeras

Each chimera (1 µg) was incubated with 10% FBS at 37 °C for different periods of time. Samples were loaded into a 2% agarose gel containing 1 μg/mL ethidium bromide. The voltage was set at 70 V, and a current was passed through the gel for 30 min. The DNA bands were visualized using Bio-Rad’s ChemiDoc MP imaging system and Image Lab software. Moreover, each chimera loaded with doxorubicin (3 µM) was incubated in 10% FBS at 37 °C to assess the intercalation stability of doxorubicin into the chimera. The fluorescence intensity of doxorubicin was measured at different time points using a Glomax microplate reader.

### 3.7. Cell Viability (MTT) Assay

MCF-7, U87, and HDF (5 × 10^3^ cells per well) were seeded in 96-well plates. After 24 h, the cells were exposed to free doxorubicin or doxorubicin loaded into chimeras at different concentrations. After 72 h of incubation at 37 °C in humidified atmosphere of 5% CO_2_, treatment was removed from the wells, followed by the addition of 15 μL MTT solution and 100 μL of the medium. After 3 h of incubation, the medium was removed, then 50 μL of DMSO was added for dissolving the formazan crystals. The absorbance was measured at a wavelength of 570 nm using a Glomax microplate reader (Promega, Madison, WI, USA). Moreover, to further validate the specific targeting of the chimeras, the cell lines were exposed to doxorubicin-loaded chimeras for only 4 h, then the treatment was removed, and 100 μL of the medium was added. After 72 h of incubation, the MTT assay was conducted as described previously.

### 3.8. Cellular Uptake Assesment of Free Doxorubicin and Doxorubicin-Loaded Chimeras Using FACS

MCF-7 and U87 (2 × 10^5^ cells per well) and HDF (1 × 10^5^ cells per well) were seeded in twelve-well plates. After culture for 24 h, the cells were treated with either 2 µM of free doxorubicin or doxorubicin-loaded chimeras, the chimeras contained 30% labeled sequences. For a negative control, cells were incubated with a complete culture medium without added chimeras. After incubation, the culture medium and treatment were removed, and the cells were washed in PBS (0.5 mL twice), then harvested using trypsin. The fluorescence intensity of doxorubicin and Cy5 (excitation wavelengths of 470 and 649 nm and emission wavelengths of 595 and 647 nm, respectively) was measured in cells using flow cytometry (FACS Canto II, BD San Diego, CA, USA).

### 3.9. Confocal Laser Microscopy

MCF-7, U87, and HDF (5 × 10^5^ cells per well) were seeded into a six-well plate containing glass coverslips and incubated for 24 h at 37 °C. The cells were then incubated with 0.6 mL of culture medium containing 2 µM of either free doxorubicin or doxorubicin loaded into fluorophore-labeled chimeras, respectively. For a negative control, cells were incubated with a complete culture medium without treatment. After incubation at 37 °C for 3 h, the cells were washed with 0.7 mL PBS, then incubated with 0.5 mL of 4% formaldehyde for 15 min at room temperature in the dark. After that, the cells were washed once with PBS, then the PBS was removed, and 300 µL of DAPI diluted 1: 1500 in PBS was added and incubated for 5 min in the dark. Finally, the cells were washed once with 0.7 mL of PBS, and the coverslips were placed face down on a glass slide with 10 µL of Fluorescence Mounting Medium (Dako Omnis), then left to dry overnight in the dark. Samples were analyzed by Zeiss LSM780 confocal microscope system (Carl Zeiss AG, Jena, Germany).

## 4. Conclusions

We generated the anti-nucleolin AS1411 aptamer-based chimera (AS1411–ATPapt), which is able to deliver doxorubicin with high efficiency into cancer cells expressing nucleolin receptors. In addition, the drug loaded in the chimera was effectively released and delivered to its correct destination in a spatiotemporally controlled style, as demonstrated by flow cytometry and confocal laser microscopy. In addition, this chimera is characterized by its simple preparation, cheap manufacturing, and good payload capacity. Integrating the targeting properties and therapeutic potential of aptamers, several drugs can be also used for the generation of multifunctional molecules in which multiple functions are combined to enhance the specificity and largely improve the effectiveness of the drugs. Overall, the AS1411–ATPapt chimera could provide a promising delivery system for doxorubicin in the future to improve clinical outcomes as well as decrease the side toxicity of doxorubicin in cancer patients.

## Figures and Tables

**Table 1:** Nucleic acid sequences.

DNA Strand	Sequence
ATP aptamer	5′-ACC TGG GGG AGT ATT GCG GAG GAA GGT-3′
AS1411–cDNA for ATP aptamer	5′ -GGTGGTGGTGGTTGTGGTGGTGGTGGTTTTTTTTTTTT ACCTTCCTCCGCAATACTCCCCCAGGT-3′
CRO–cDNA for ATP aptamer	5′ -CCTCCTCCTCCTTCTCCTCCTCCTCCTTTTTTTTTTTTT ACCTTCCTCCGCAATACTCCCCCA GGT -3′
AS1411–cDNA for ATP aptamer—Iowa Black RQ	5′-GGTGGTGGTGGTTGTGGTGGTGGTGGTTTTTTTTTTTT ACCTTCCTCCGCAATACTCCCCC AGGT-Iowa Black RQ quencher 3′
Cy5–ATP aptamer	5′-cy5-ACC TGG GGG AGT ATT GCG GAG GAA GGT-3′
CRO–cDNA for ATP aptamer— Iowa Black RQ	5′-CCTCCTCCTCCTTCTCCTCCTCCTCCTTTTTTTTTTTTT ACCTTCCTCCGCAATACTCCCCCAGGT Iowa Black RQ quencher -3′

## Data Availability

Not applicable.
